# Comparison of Serum Adropin Levels in Patients with Diabetes Mellitus, COVID-19, and COVID-19 with Diabetes Mellitus

**DOI:** 10.5152/eurasianjmed.2022.22128

**Published:** 2022-06-01

**Authors:** Pelin Aydın, Sevgi Karabulut Uzunçakmak, İbrahim Hakkı Tör, Arzu Bilen, Ayşe Özden

**Affiliations:** 1 Department of Anesthesiology and Reanimation, Erzurum Regional Training and Research Hospital, Erzurum, Turkey; 2 Department of Health Services, Bayburt University Vocational School, Bayburt, Turkey; 3 Department of Internal Medicine, Atatürk University Faculty of Medicine, Erzurum, Turkey; 4 Department of Pediatric Endocrinology, Erzurum Regional Training and Research Hospital, Erzurum, Turkey

**Keywords:** Adropin, COVID-19, DM, d-dimer, CRP

## Abstract

**Objective:** In the present study, the relationship between a poor prognosis and adropin levels in diabetic patients with coronavirus disease 2019 was investigated by measuring serum adropin levels and levels of D-dimer, C-reactive protein, and ferritin, which are considered prognostic factors for coronavirus disease 2019.

**Materials and Methods:** Hundred volunteer participants treated in the Erzurum Regional Training and Research Hospital were included in this study. Serum adropin levels were measured by enzyme-linked ­immunosorbent assay. The relationship between serum adropin level and C-reactive protein, ferritin, and D-dimer levels was analyzed by correlation analysis.

**Results:** The participants’ serum adropin levels differed between the groups (*P* = .0007). The control group had the highest adropin levels among groups. The lowest adropin levels were in the COVID + diabetes mellitus group. Adropin levels of diabetes mellitus, COVID, and diabetes mellitus + COVID groups were significantly decreased when compared to the control (*P* < .05). There was a significant negative correlation between adropin and C-reactive protein, D-dimer, and ferritin.

**Conclusion:** Adropin can be used as an auxiliary biomarker, a prognostic factor in the early management of coronavirus disease 2019 patients with diabetes mellitus. We think that our study will guide future studies conducted in this field.

## Introduction

Coronavirus disease 2019 (COVID-19) is an infectious respiratory disease caused by the betacoronavirus severe respiratory coronavirus syndrome coronavirus 2 (SARS-CoV-2), which originated in Wuhan, China.^1^ The COVID-19 outbreak was declared a pandemic by the World Health Organization on March 11, 2020, due to its rapid progress in a very short time, affecting large numbers of people and causing deaths.^2^

Severe respiratory coronavirus syndrome coronavirus 2 infection can be asymptomatic or accompanied by symptoms of a viral respiratory infection, such as fever, cough, weakness, and dyspnea. In severe cases, pneumonia, severe acute respiratory syndrome, heart failure, kidney failure, and even death may occur.^3^ The main causes of death related to COVID-19 are respiratory failure, septic shock, kidney failure, bleeding, and heart failure.^4^ When the case series was examined, it was determined that arterial hypertension, coronary heart disease, obesity, and diabetes were the risk factors of COVID-19 itself. However, it has been reported that COVID-19 has a more severe clinical course and the mortality rate is higher in these patients.^5,6^

Diabetics make up a significant proportion of COVID-19 patients hospitalized.^7^ It is observed that among COVID-19 patients admitted to intensive care units, those with underlying diabetes mellitus (DM) are also common.^8,9^ These patients are at high risk for COVID-19 and have a worse prognosis than those who do not have diabetes.^10^ The pathophysiology underlying the severe course of COVID-19 infection and the increased risk of respiratory failure and death in patients with diabetes is not yet fully understood, but various mechanisms are thought to be responsible. Endothelial damage due to inflammation, oxidative stress, and cytokine production, an increased risk of complications due to DM, and an increased risk of damage to vital organs are suggested mechanisms.^11,12^ One of the molecules known to be associated with endothelial damage is the hormone adropin.

Adropin is a peptide that regulates glycolipid metabolism. It is encoded by the energy homeostasis-associated gene. It is expressed in the heart and gastrointestinal tract, but mainly in the liver and brain. It is also known to be present in the human circulatory system.^13^ In recent studies, it has been found that adropin has a beneficial effect on glucose homeostasis and dyslipidemia. It has also been shown to have beneficial effects in obesity-related hyperinsulinemia and in improving energy homeostasis.^14^ It has been shown that serum adropin levels decrease in many diseases such as coronary atherosclerosis, hypertension, diabetic nephropathy, and polycystic ovary disease.^15,16^ Clinical studies have confirmed that serum adropin levels are negatively correlated with the risk factors of metabolic diseases. It was found that low levels of adropin are associated with the development of the metabolic syndrome. It has been stated that adropin is a potentially protective agent against the development of the metabolic syndrome.^17^ The relationship between adropin levels and the development of atherosclerosis was investigated in patients with and without type 2 DM. Adropin levels were found to be low in patients with type 2 DM. At the same time, it was reported that adropin levels are inversely proportional to the severity of coronary atherosclerosis.^18^ It is known that adropin is involved in the regulation of endothelial function.^19^ There is an association between decreases in its level and endothelial dysfunction and metabolic syndrome.^20^ Similarly, it has been stated that low circulating adropin levels in patients with type 2 DM are a risk factor for endothelial dysfunction.^21^

Although it has been shown that endothelial dysfunction is one of the major underlying causes of advanced courses of COVID-19 infection in DM patients, respiratory failure, and increased risk of death, its relationship with serum adropin levels, which is an indicator of endothelial dysfunction, has not yet been studied. In the present study, the relationship between a poor prognosis and adropin levels in diabetic patients with COVID was investigated by measuring serum adropin levels and levels of D-dimer, C-reactive protein (CRP), and ferritin, which are considered prognostic factors for COVID-19.

## Materials and Methods

### Study Design

Hundred volunteer participants treated in Erzurum Regional Training and Research Hospital were included in this study. All participants were tested for COVID-19 via real-time polymerase chain reaction (RT-PCR). The number of participants was determined by performing a g-power analysis. The participants included 25 healthy controls (COVID-19 negative), 25 patients with DM (COVID-19 negative), 25 patients with COVID-19 without DM (COVID-19 positive), and 25 patients with COVID-19 infection and DM (COVID-19 positive). COVID-19 patients and COVID-19 and DM patients had positive COVID-19 RT-PCR test results. COVID-19 patients with goiter, gout, hypertension, congestive heart failure, coronary artery disease, atrial fibrillation, chronic kidney failure, and cancer were excluded. Diabetes mellitus patients with coronary artery disease, hypothyroid, atrial fibrillation, and chronic kidney failure were excluded from the study. However, the included DM patients have comorbid diseases such as hyperlipidemia, depression, Parkinson’s disease, and dry eye syndrome. Diabetes mellitus patients used oral antidiabetic or insulin. Patients diagnosed with type 2 DM, who achieved glycemic stability after starting diabetes treatment, were included in the study (hemoglobin A1c ≤7.0%). This study was approved by the Ethics Committee of Atatürk University School of Medicine (ethic number: February 24, 2022/B.30.2.ATA.0.01.00/208)) and was performed in accordance with the Helsinki Declaration. All participants were educated about the aim of the study and written informed consent was obtained from all participants. Demographic and clinical data were obtained from hospital records and analyzed retrospectively. Laboratory test results of CRP, ferritin, and D-dimer were also obtained from hospital records.

### Sample Collection

Blood samples were collected in tubes containing non-ethylenediamine tetraacetic acid. Blood samples were centrifuged at +4 °C, 4000 rpm for 10 minutes. Serum samples were separated from the tubes and stored at −80 °C until use.

### Measurement of Serum Adropin Level

Serum adropin levels were measured via a commercial enzyme-linked immunosorbent assay according to the manufacturer’s instructions (Bioassay Technology Laboratory, Wuhan, China). The reference range of 5-10 000 ng/L has been considered for adropin.

### Statistical Analysis

#### Sample Size Calculation and Power Analysis

The data included in the study were analyzed using the GraphPad program and had an observation power of 95 % at the alpha = 0.05 significance level. It would be necessary to have approximately 25 patients per group to obtain a significant statistical value. It was seen that 100 patients included in the study were sufficient for statistical analysis.

Data were analyzed with GraphPad Prism 5. The variables were evaluated using Kolmogorov–Smirnov or Shapiro–Wilk tests to determine whether or not they were normally distributed. Kruskal–Wallis was used to compare the variables that were not normally distributed and the Dunns test was utilized also as a post hoc test. To test the differences between 2 groups for non-normally distributed data, Mann–Whitney *U*-test was used. Spearman correlation was used to assess the correlation between serum adropin level and CRP, D-dimer, and ferritin levels. *P* values ≤.05 were considered statistically significant.

## Results

### Characteristics of Participants

Totally 100 participants were enrolled in the study. In the study, 40% (n = 40) of the participants were male and 60% (n = 60) were female. The age range was 23-70 in DM patients, 23-87 in COVID patients, 38-92 in COVID+DM patients, and 31-75 in healthy individuals. Liver function tests and kidney function tests were not performed because they were not directly related to the study. Forty-two percent (n = 21) of the patients (COVID, COVID+DM) infected with COVID died. Of the total number of COVID-19 patients (COVID, COVID+DM), 48% (n = 24) needed mechanical ventilation.

### Laboratory Findings of Patients

Patients have been separated into 4 groups as in Table 1. The first group (control) consists of healthy control, the second group (DM) is just patients with DM, the third group consists of patients who are infected with SARS-CoV-2, and the fourth group consists of patients with DM who are infected by SARS-CoV-2. C-reactive protein, D-dimer, ferritin, and adropin levels of groups are summarized in Table 1. The COVID+DM patients showed higher CRP levels than other patients. D-dimer values of COVID+DM patients were higher than COVID patients and healthy control. The highest ferritin level was seen in COVID patients. Adropin level was decreased in COVID+DM patients compared with other groups.

### Serum Adropin Levels of Participants

**Figure 1.**
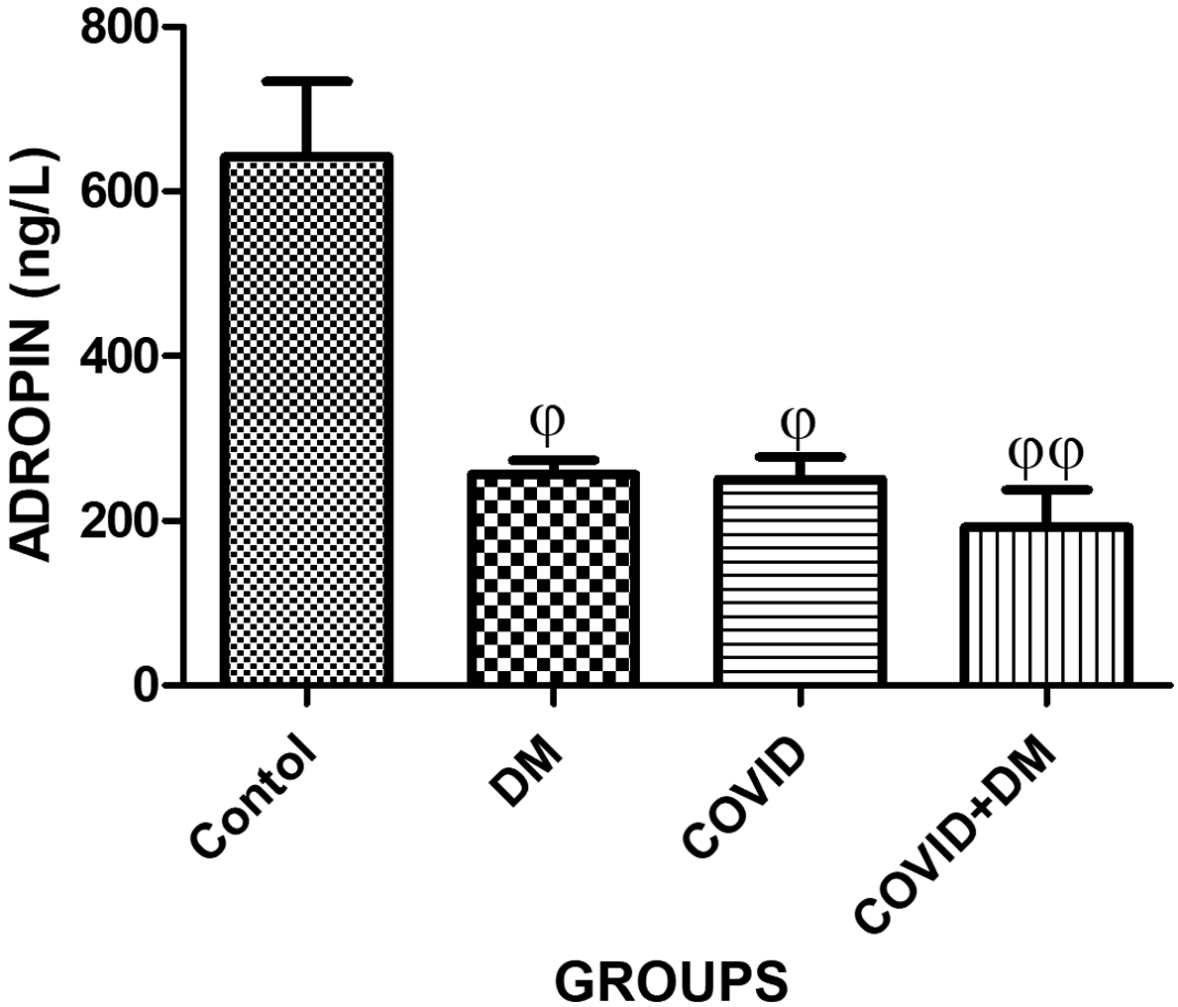
Comparison of serum adropin levels in participants. COVID, patients infected with SARS-CoV-2; DM, diabetes mellitus; COVID+DM, patients with DM who is infected with SARS-CoV-2, φ, according to the control group.

The participants’ serum adropin levels differed between the groups (Figure 1) (*P* = .0007). The control group had the highest adropin levels among the groups. The lowest adropin levels were in the COVID+DM group. Adropin levels of DM, COVID, and DM+COVID groups were significantly decreased when compared to control (*P* < .05).

### Correlation Analysis of Adropin

We investigated the relation of serum adropin, CRP, D-dimer, and ferritin levels in patients. The results of the correlation analysis of adropin and serum inflammatory markers are summarized in Table 2. D-dimer values of DM patients were not accessible, so their D-dimer values were excluded from the analysis. There was a significant negative correlation between adropin and CRP, D-dimer, and ferritin (*r* = −0.3219 *P* = .0311; *r* = −0.3988 *P* = .0263; *r* = −0.3691 *P* = .0226, respectively).

## Discussion

As a result of our study, we found that adropin, which has been previously shown to be associated with endothelial damage in different studies in the literature, was significantly lower in DM patients with COVID-19. At the same time, we observed a negative correlation between the levels of D-dimer, ferritin, and CRP, known to be prognostic factors for COVID-19, and serum adropin levels in the present study. We think that low adropin levels in DM patients, which we have also shown in the present study and which are also reported in the literature, may have worsened the course of the disease by further increasing the endothelial damage already caused by COVID.

Diabetes mellitus has been one of the most important risk factors for higher severity of disease in patients with COVID-19. The fact that people with diabetes have an innate and adaptive immune response that is irregular makes them more susceptible to inflammatory processes and cytokine storms. At the same time, people with diabetes are also at a higher risk of thrombotic events due to an imbalance between coagulation factors and fibrinolysis.^22^ One of the molecules thought to be related to the chronic inflammatory process in DM patients is the hormone adropin. It appears that studies on adropin are more focused on metabolic and cardiovascular diseases. In the studies conducted, the role of adropin in regulating metabolism and improving the functions of endothelial cells is noted. However, it is thought that adropin itself also has immunological effects. Therefore, recently, the relationship between adropin hormone levels and inflammation has become one of the topics that scientists are focusing on and its connection with various signaling pathways has been studied. It has been reported that adropin has potential anti-inflammatory effects and may improve the inflammatory response in various disease processes.^23^ It has been shown that adropin can modulate PPAR-γ expression, which is involved in many important regulatory pathways including lipid and glucose homeostasis, cell differentiation, proliferation, apoptosis, and inflammation.^13^ It is known that macrophage infiltration can be reduced and inflammation will be alleviated by PPAR-γ activation. In 1 study, it was shown that adropin upregulates PPAR-γ expression, and this modulation contributes to the regulation of the inflammatory process by acting on the anti-inflammatory or proinflammatory phenotypes of macrophages. In the present study, the release of tumor necrosis factor-alpha and interleukin 6 (IL-6) with proinflammatory effects was also inhibited by adropin. As a result, inflammation decreased and anti-atherosclerotic effects occurred.^24^ In our study, serum adropin levels were significantly lower in the COVID+DM patients than in the other groups. However, the need for mechanical ventilation and the mortality rate were higher in these patients. Decreased adropin levels in DM patients may have prevented the emergence of an adequate anti-inflammatory effect after these patients were infected with COVID. Initially, inflammation increased in these patients with already low levels of adropin, there was no adequate anti-inflammatory response to COVID-19, and thus we think that the clinical course may have been further aggravated. There are several studies in the literature that show that adropin promotes endothelial homeostasis. The endothelium is very important in maintaining vascular homeostasis. The resulting dysfunction of the endothelium leads to increased vascular permeability and aggravation of inflammation. This dysfunction is closely related to DM, hypertension (HT), and atherosclerotic heart diseases.^25^ It has been reported that adropin reduces endothelial permeability and prevents the passage of macrophages in response to inflammatory stimuli, ultimately reducing inflammation.^19^ In our study, serum adropin levels, which were initially low in the DM patients, may have decreased further with the addition of COVID-19 and increased endothelial damage. Increased endothelial damage may also have led to an increase in the severity of inflammation and, as a result, an increase in mortality.

In order to better understand the relationship between adropin, COVID-19, and DM, we also analyzed the correlation between serum adropin levels and CRP (an inflammatory biomarker), D-dimer, and ferritin. It has been reported that biomarkers such as CRP, ferritin, lactate dehydrogenase, D-dimer, IL-6, and fibrinogen may give an idea about the course of the disease in COVID-19 patients.^26^ C-reactive protein, an acute-phase protein, is synthesized by the liver in response to IL-6 and is a widely used biomarker of inflammation. It is indicative of systemic inflammation and severe infection.^27^ The relationship between CRP and COVID-19 has been examined in many studies. A relationship was reported between increased CRP concentrations and higher disease severity in COVID-19, and CRP was considered a prognostic factor.^28^ High levels of CRP in the early stage of COVID-19 have been associated with lung damage and the severity of the disease.^29^ It has been reported that D-dimer levels are higher in severe COVID-19 patients; the mechanism of the D-dimer increase in these patients is not yet understood, but D-dimer will be useful in the early diagnosis of severe disease.^30^ In a similar study, a higher level of D-dimer was measured in patients with severe COVID-19 than in non-severe patients, and it was concluded that the level of D-dimer was associated with COVID-19 severity.^31^ Another biomarker that is a bad prognosis sign, like D-dimer in COVID-19, is the ferritin value. High ferritin values have also been associated with a poor prognosis in COVID-19.^32^ In our study, we found a negative correlation between serum adropin levels and CPR, D-dimer, and ferritin levels. C-reactive protein, D-dimer, and ferritin levels increased with decreasing adropin levels. This may have increased the need for ventilation in COVID-19 patients with DM, leading to a worse prognosis and risk of death. We think that a low level of adropin exacerbates endothelial damage, which can lead to a severe course of inflammation and an increased risk of thromboembolism.

In conclusion, in the present study, we found that adropin levels, which are lower in DM, were much lower in patients with DM and COVID. At the same time, there was a negative correlation between adropin levels and CRP, D-dimer, and ferritin levels, which are considered prognostic for COVID-19. Adropin can be used as an auxiliary biomarker, a prognostic factor in the early management of COVID-19 patients with DM. We think that our study will guide future studies conducted in this field.

## Figures and Tables

**Table 1.** Comparison of Laboratory Findings of Patients

Parameters	Control (Mean ± SD)	DM (Mean ± SD)	COVID (Mean ± SD)	COVID+DM (Mean ± SD)	*P*
CRP (mg/L)	1.861 ± 2.78	12.57 ± 17.84	42.28 ± 32.75	68.28 ± 72.91	**.0001** ***
D-dimer (µg/mL)	354.1 ± 103	-	1667 ± 1827	3067 ± 7910	**.0004** ***
Ferritin (ng/mL)	113.5 ± 36.2	117.5 ± 143.7	460 ± 597.9	438 ± 458.4	**.0066** **
Adropin (ng/L)	642.6 ± 387	256.1 ± 70.85	367.3 ± 179.7	162.4 ± 115.1	**.0007** ***

Kruskal–Wallis test was used to compare the groups.

D-dimer values of DM patients were not available

Bold *P* values were statically significant.

CRP, C-reactive protein; DM, diabetes mellitus; SD, standard deviation.

**Table 2.** Correlation Analysis of Serum Inflammatory Markers and Adropin

**Spearman Correlation**		
	**r Value**	**P**
**CRP** (mg/L)	−0.3219	**.0311** *
**D-dimer** (µg/mL)	−0.3988	**.0263** *
**Ferritin** (ng/L)	−0.3691	**.0226** *

Spearman correlation was used.

Bold *P* values were statically significant.

CRP, C-reactive protein.
